# VPS13A is closely associated with mitochondria and is required for efficient lysosomal degradation

**DOI:** 10.1242/dmm.036681

**Published:** 2019-02-22

**Authors:** Sandra Muñoz-Braceras, Alba R. Tornero-Écija, Olivier Vincent, Ricardo Escalante

**Affiliations:** Instituto de Investigaciones Biomédicas Alberto Sols, Department of Experimental Models of Human Diseases, Consejo Superior de Investigaciones Científicas (CSIC)/Universidad Autónoma Madrid (UAM), 28029-Madrid, Spain

**Keywords:** Autophagy, Chorea-acanthocytosis, Chorein, Lysosomes, Membrane contact sites, VPS13A

## Abstract

Members of the VPS13 family are associated with various human diseases. In particular, the loss of function of VPS13A leads to chorea-acanthocytosis (ChAc), a rare neurodegenerative disease without available curative treatments. Autophagy has been considered a promising therapeutic target because the absence of VPS13A causes a defective autophagy flux. However, the mechanistic details of this deficiency are unknown. Here, we identified Rab7A as an interactor of one of the VPS13 family members in *Dictyostelium discoideum* and showed that this interaction is conserved between the human homologs VPS13A and RAB7A in HeLa cells. As RAB7A is a key player in endosome trafficking, we addressed the possible function of VPS13A in endosome dynamics and lysosome degradation. Our results suggest that the decrease in autophagy observed in the absence of VPS13A may be the result of a more general defect in endocytic trafficking and lysosomal degradation. Unexpectedly, we found that VPS13A is closely localized to mitochondria, suggesting that the role of VPS13A in the endolysosomal pathway might be related to inter-organelle communication. We show that VPS13A localizes at the interface between mitochondria-endosomes and mitochondria-endoplasmic reticulum and that the presence of membrane contact sites is altered in the absence of VPS13A. Based on these findings, we propose that therapeutic strategies aimed at modulating the endolysosomal pathway could be beneficial in the treatment of ChAc.

This article has an associated First Person interview with the first author of the paper.

## Abstract (precis)

**Summary:** VPS13A, which is implicated in chorea-acanthocytosis, is essential for efficient lysosomal degradation, while localized in close association with mitochondria. We propose that inter-organelle communication may be relevant in the pathogenesis of the disease.

## INTRODUCTION

Chorea-acanthocytosis (ChAc) is a rare disease that leads to progressive neurodegeneration. Movement disorders such as chorea, dystonia or parkinsonism, and the presence of acanthocytes (misshapen red blood cells) are characteristic of this fatal disease. To date, therapeutic approaches are based on a few published empirical observations and aim only at mitigating symptoms (Walker, 2015a). Therefore, any finding on the molecular etiology of ChAc could have important implications for the development of effective treatments to cure or slow down the progression of this disease.

Mutations in the vacuolar protein sorting 13 A (*VPS13A*) gene, which mostly lead to the absence of the protein (also known as chorein), are the cause of ChAc (Dobson-Stone et al., 2002, 2004; Rampoldi et al., 2001; Tomiyasu et al., 2011; Ueno et al., 2001). The other three members of the human VPS13 family are also involved in disease. Mutations in *VPS13B* lead to Cohen syndrome (Kolehmainen et al., 2003); mutations in *VPS13C* have been identified as a cause of an autosomal-recessive, early-onset and severe form of Parkinson's disease (Lesage et al., 2016; Schormair et al., 2018); and, most recently, mutations in *VPS13D* have been linked to other movement disorders (Gauthier et al., 2018; Seong et al., 2018). In addition, genomic data have identified *VPS13A-D* variants in other neurological disorders (Fromer et al., 2014; McCarthy et al., 2014; Meda et al., 2012), in various types of cancer (An et al., 2012; Furukawa et al., 2011; Morisaki et al., 2014; Park et al., 2016b; Yang et al., 2016b) and in diabetes (Grarup et al., 2011; Saxena et al., 2010; Strawbridge et al., 2011; Windholz et al., 2011).

VPS13 proteins are very large proteins that share conserved domains or structural features. They are widely conserved during eukaryotic evolution, from unicellular organisms to humans (Velayos-Baeza et al., 2004), so their study can be addressed in different models (Rzepnikowska et al., 2017). In *Saccharomyces cerevisiae*, only one Vps13 protein is present, but it participates in several cellular processes such as protein trafficking, vesicular transport and fusion (Brickner and Fuller, 1997; De et al., 2017; Redding et al., 1996), sporulation (Park and Neiman, 2012), mitochondrial homeostasis (Park et al., 2016a) and functional compensation of the loss of endoplasmic reticulum (ER)-mitochondria encounter structure (ERMES), which are membrane contact sites (MCS) between the mitochondria and the ER (Lang et al., 2015; Park et al., 2016a; Xue et al., 2017). In other model organisms, such as *Tetrahymena thermophila*, *Dictyostelium discoideum* and *Drosophila melanogaster*, several VPS13 proteins are present and have been implicated in different cellular processes such as phagocytosis (Leiba et al., 2017; Muñoz-Braceras et al., 2015; Samaranayake et al., 2011), autophagy (Muñoz-Braceras et al., 2015) and mitochondrial homeostasis (Anding et al., 2018). The functions attributed to the human VPS13 proteins are particularly diverse, especially for VPS13A, which has been reported to be involved in cytoskeleton organization (Alesutan et al., 2013; Föller et al., 2012; Honisch et al., 2015a; Schmidt et al., 2013; Stanslowsky et al., 2016), phosphoinositide regulation (Park et al., 2015), tyrosine phosphorylation dependent on LYN (De Franceschi et al., 2011), secretion (Honisch et al., 2015b; Schmidt et al., 2013), calcium homeostasis (Yu et al., 2016) and autophagy (Lupo et al., 2016; Muñoz-Braceras et al., 2015). VPS13B is required for the organization of the Golgi apparatus and protein glycosylation (Duplomb et al., 2014; Seifert et al., 2011), VPS13C for adipogenesis and lipolysis (Ramseyer et al., 2018; Yang et al., 2016a), and both VPS13C and VPS13D for the maintenance of mitochondrial homeostasis (Anding et al., 2018; Lesage et al., 2016). Despite the growing evidence of the involvement of VPS13 proteins in many cellular processes, the mechanism by which their alteration leads to different human diseases and the relative importance of each process in the pathogenesis of these diseases are still unknown.

Macroautophagy, hereafter referred to as autophagy for simplicity, is a cellular degradation process essential for the maintenance of cellular homeostasis. Its proper functioning is particularly crucial for neuronal survival and has therefore been implicated in several neurodegenerative diseases (Kiriyama and Nochi, 2015). In addition, autophagy is necessary during erythropoiesis (Vessoni et al., 2012). We previously reported a partial defect in autophagy in HeLa cells lacking VPS13A (Muñoz-Braceras et al., 2015) and hypothesized that this defect could play an important role in the etiology of ChAc. A deficiency of autophagy during this disease was supported by the work of Lupo and colleagues, who described a delayed autophagic clearance during erythroid maturation in patients (Lupo et al., 2016). In addition, increased levels of the autophagic proteins ULK1, ATG13, ATG7 and LC3-I were observed in ChAc erythrocyte lysates (Lupo et al., 2016), but the exact mechanism leading to defective autophagy remains unknown.

In this study, we aimed to investigate in more detail the role of VPS13A in autophagy by first using *D. discoideum* as a model organism and then human cells. Our results suggest that the defects observed in autophagy in the absence of VPS13A are most likely the consequence of a more general impairment of the endolysosomal pathway. In addition, we investigated the subcellular localization of VPS13A and found an unexpected predominant localization to mitochondria, which provides valuable insight into the possible mechanisms by which the absence of VPS13A may lead to lysosomal dysfunction.

## RESULTS

### RAB7A interacts with *D. discoideum* TipC and human VPS13A

Our previous study of a member of the VPS13 family, TipC, in *D. discoideum* provided the first evidence of VPS13 proteins involvement in autophagy. The *tipC^−^* mutant presents a multitipped phenotype, which is a characteristic developmental phenotype of autophagy mutants in this social amoeba (Mesquita et al., 2015; Otto et al., 2003, 2004; Tung et al., 2010), and, accordingly, this mutant exhibits impaired autophagy along with additional defects in sporulation and phagocytosis. We found that these phenotypes were largely rescued by the overexpression of the C-terminal region of TipC (amino acids 2725-3848), which contains conserved domains found in virtually all VPS13 proteins, including human VPS13A. In addition, we demonstrated that autophagy is impaired in VPS13A-depleted human HeLa cells (Muñoz-Braceras et al., 2015). Based on these results, we hypothesized that the C-terminal region of TipC in *D. discoideum* could mediate its interaction with proteins involved in the execution or regulation of autophagy and that this interaction could be conserved for human VPS13A. Therefore, in the present study, we used *D. discoideum* as a starting point to shed light on the molecular function of VPS13 proteins.

**Fig. 1.**
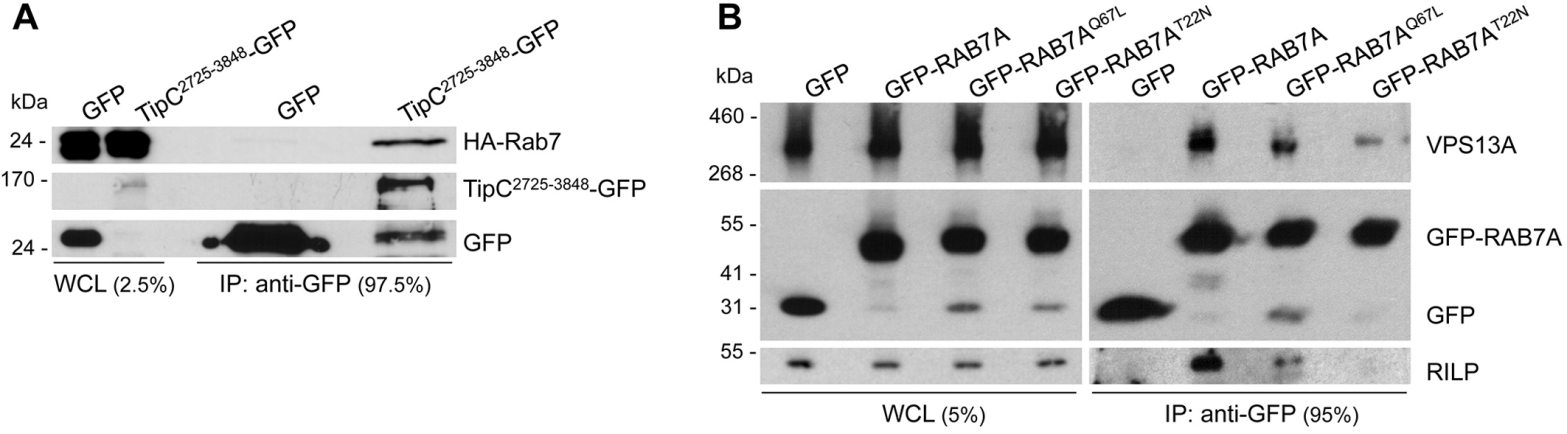
***Dictyostelium discoideum* TipC and human VPS13A co-immunoprecipitate with Rab7.** (A) The C-terminal region of TipC (amino acids 2725-3848) fused to GFP was immunoprecipitated from lysates of the *D. discoideum tipC^−^* mutant overexpressing this polypeptide and HA-Rab7A. The immunoprecipitates were analyzed by western blotting using an anti-HA antibody and an anti-GFP antibody. HA-Rab7A co-immunoprecipitated with TipC^2725-3848^-GFP but not with GFP alone, which was immunoprecipitated as a control. (B) GFP-RAB7A was immunoprecipitated from lysates of HeLa cells transiently transfected with wild-type GFP-RAB7A, constitutively active GFP-RAB7A^Q67L^ and constitutively inactive GFP-RAB7A^T22N^. GFP was included as a negative control. The immunoprecipitates were analyzed by western blotting using an anti-VPS13A antibody, an anti-GFP antibody and an anti-RILP antibody. The percentage of sample loaded is indicated for whole-cell lysates (WCL) and immunoprecipitates (IP). Images are representative of three (A) and two (B) independent experiments.

We used liquid chromatography (LC) coupled to tandem mass spectrometry (MS/MS) to identify proteins that co-immunoprecipitate with *D. discoideum* TipC^2725-3848^-GFP and not with a control GFP (Table S1). One of the possible interactors identified was Ras-like in rat brain 7A (Rab7A), a protein involved in autophagy and phagocytosis in *D. discoideum* and other organisms (Guerra and Bucci, 2016; Rupper et al., 2001). The interaction was confirmed by pulldown experiments using *D. discoideum* cells expressing hemagglutinin (HA)-tagged Rab7A and TipC^2725-3848^-GFP (Fig. 1A). We then analyzed the interaction of the corresponding human proteins in HeLa cells transfected with GFP-tagged wild-type or mutant constitutively active (GTP-bound) or constitutively inactive (GDP-bound) forms of the RAB7A GTPase. We observed that endogenous VPS13A specifically co-immunoprecipitated with GFP-RAB7A, and that VPS13A interacts more with the constitutively active RAB7A mutant than with the constitutively inactive form of the GTPase (Fig. 1B), similarly to Rab-interacting lysosomal protein (RILP), which is a well-known effector of RAB7A (Cantalupo et al., 2001). These results suggest that the ability to interact with RAB7A is conserved among the VPS13 proteins and lead to the hypothesis that VPS13 proteins may participate in autophagy through their interaction with RAB7A.

### The absence of VPS13A has a broad impact on the autophagic and endolysosomal pathway

RAB7A is recruited to endocytic vesicles during their maturation and it is a key participant in the endolysosomal pathway. Its roles in autophagy and the endocytic pathway involve similar processes, as it regulates the movement and maturation of both autophagic and endocytic vesicles and their fusion with lysosomes (Guerra and Bucci, 2016). Moreover, there is a close relationship between the autophagic and endocytic pathways, as autophagosomes can fuse with endosomes to form amphisomes prior to fusion with lysosomes. Therefore, if there is a functional link between VPS13A and RAB7A, *VPS13A* knockdown may cause alterations of RAB7A itself and the endocytic pathway. Knockdown of *VPS13A* was performed by RNA interference using two different small interfering RNAs (siRNAs) to rule out possible off-target effects.

**Fig. 2.**
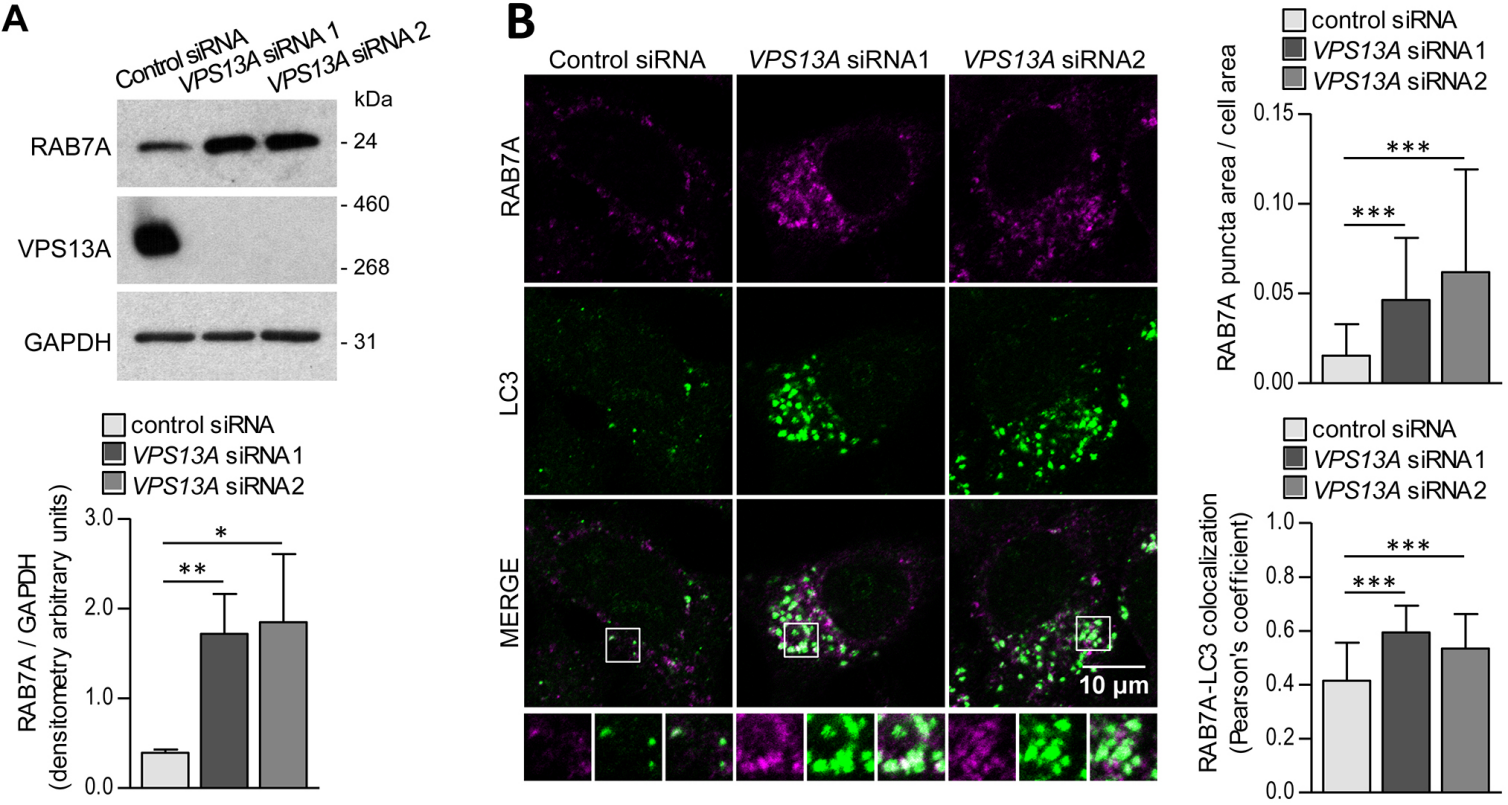
**RAB7A is accumulated and clustered in a juxtanuclear region in VPS13A-depleted cells colocalizing with LC3.** (A) HeLa cells were treated with control or *VPS13A* siRNAs and the level of RAB7A in the lysates was assessed by western blotting. The absence of VPS13A in the *VPS13A* siRNA-treated cells was assessed using an anti-VPS13A antibody. Protein bands of three independent experiments were densitometered and normalized against GAPDH as a loading control. (B) RAB7A and LC3 were observed by co-immunofluorescence of control and *VPS13A* siRNA-treated cells. Enlargements of selected areas are shown. The area of the RAB7A-positive vesicles was measured and normalized against the total cell area to obtain a quantitative index of the accumulation of RAB7A. The colocalization of RAB7A with LC3 was also quantified. Three independent experiments were performed and, on average, 17 cells were quantified per experiment and per condition. Means±s.d. are plotted (*P<0.05, **P<0.01, ***P<0.001).

First, we evaluated the levels and distribution of RAB7A in the absence of VPS13A. Interestingly, we found a prominent accumulation of endogenous RAB7A in protein lysates of VPS13A*-*depleted cells (Fig. 2A). This accumulation was also obvious from the confocal microscopy visualization of the protein, which revealed that endogenous RAB7A was notably accumulated in vesicles in a juxtanuclear region of the *VPS13A* siRNA-treated cells (Fig. 2B). In previous work, we observed a similar pattern of accumulation of microtubule-associated protein 1 light chain 3B (LC3B; also known as MAP1LC3B), a marker of autophagosomes, in a juxtanuclear region of the cell when *VPS13A* expression was inhibited (Muñoz-Braceras et al., 2015). We detected extensive colocalization of LC3 with RAB7A, and the quantitative assessment of the relative colocalization of both markers revealed a higher Pearson's coefficient of colocalization in VPS13A-depleted cells (Fig. 2B).

**Fig. 3.**
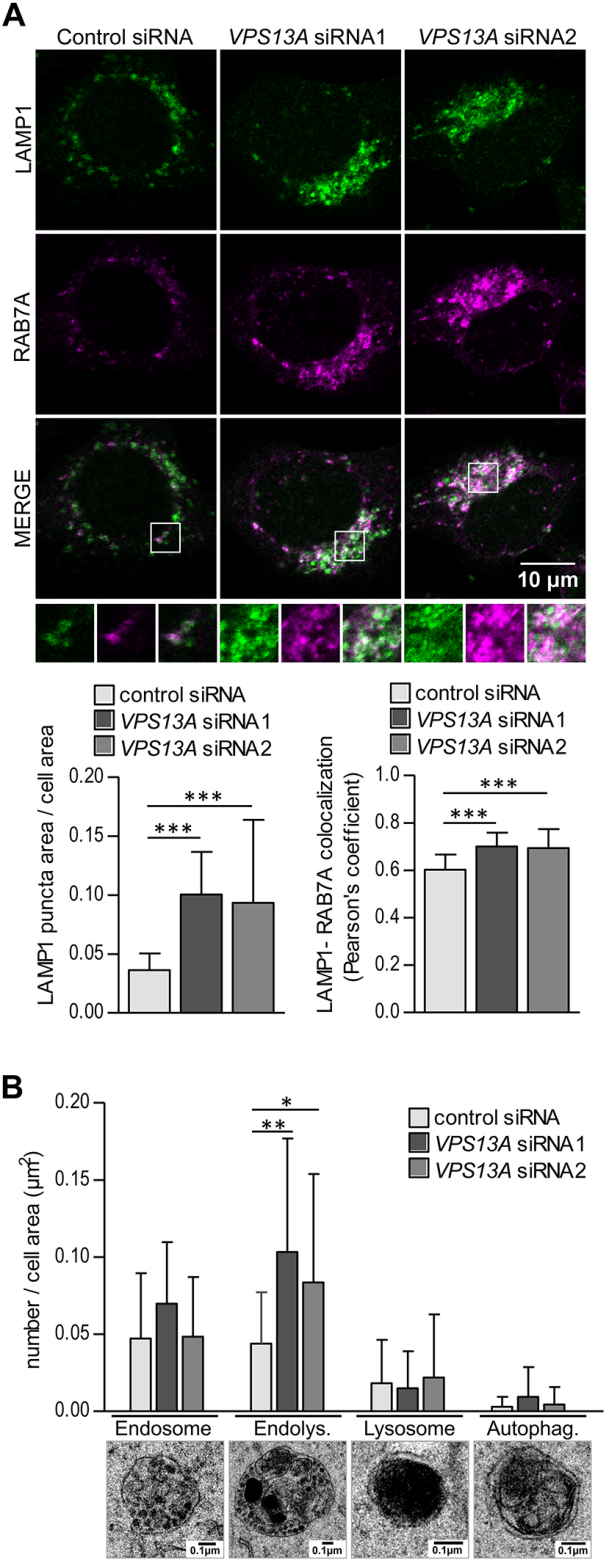
**LAMP1 and RAB7A positive vesicles accumulated in a juxtanuclear region in VPS13A-depleted cells are endolysosomes.** (A) LAMP1 and RAB7A were visualized by immunofluorescence and confocal microscopy observation of control and *VPS13A* siRNA-treated HeLa cells. Enlargements of selected areas are shown. The accumulation of LAMP1-positive vesicles and the colocalization of LAMP1 with RAB7A in three independent experiments were quantified from an average of 19 cells per condition and experiment. (B) HeLa cells treated with control or *VPS13A* siRNAs were observed by TEM. Endosomes, endolysosomes, lysosomes and autophagosomes (an example of each is shown) were identified and quantified in 20 random cell sections per sample. Means±s.d. are plotted (*P<0.05, **P<0.01, ***P<0.001).

Both autophagosomes and endosomes fuse with lysosomes for degradation of their cargo and therefore the accumulation of autophagic and endosomal markers may be due to a reduced number of lysosomes. However, lysosome-associated membrane protein 1 (LAMP1), a lysosomal marker, accumulated similarly in a juxtanuclear area in *VPS13A* siRNA-treated cells, and clearly colocalized with RAB7A (Fig. 3A) and LC3 (Fig. S1). Lysosomes are normally concentrated in a juxtanuclear region (Pu et al., 2016), to which late endosomes and autophagosomes are transported to promote fusion with lysosomes. The clustering of RAB7A and LC3 together with LAMP1 in the absence of VPS13A shows that endosomes and autophagosomes correctly reach the lysosomes, but their accumulation indicates that there is a defect in the fusion process or in the efficiency of degradation. Although higher Pearson's colocalization coefficients could be indicative of fusion, they may also be the result of pronounced accumulation of markers in the cluster, and therefore we cannot rule out the existence of a slight fusion defect that would not be detectable by conventional confocal microscopy. We then performed transmission electron microscopy (TEM) to better define the identity of the vesicles. Endosomes are single-membrane vesicles with a variable number of intraluminal vesicles and a rather electron-lucent content that distinguishes them from endolysosomes, characterized by the presence of heterogeneous and electron-dense material. Amphisomes and autolysosomes are included in the same category as endolysosomes, as they are also single-membrane vesicles with a heterogeneous content partially degraded and electron dense. Autophagosomes, on the other hand, have an intact double membrane and a non-degraded heterogeneous cytoplasmic content (Huotari and Helenius, 2011; Klumperman and Raposo, 2014). Based on these criteria, few autophagosomes were detected and no significant changes in their number were found upon *VPS13A* knockdown. Similarly, no differences in the number of endosomes were observed. In contrast, depletion of VPS13A resulted in the accumulation of endolysosomes containing electron-dense and partially degraded material (Fig. 3B). Together, these data indicate that fusion between autophagosomes, endosomes and lysosomes is not severely altered in *VPS13A* siRNA-treated cells and suggest that, although degradation should occur to some extent given the presence of endolysosomes containing electron-dense material, the blockage of autophagy in the absence of VPS13A occurs at the level of degradation.

### The absence of VPS13A leads to defective lysosomal degradation

**Fig. 4.**
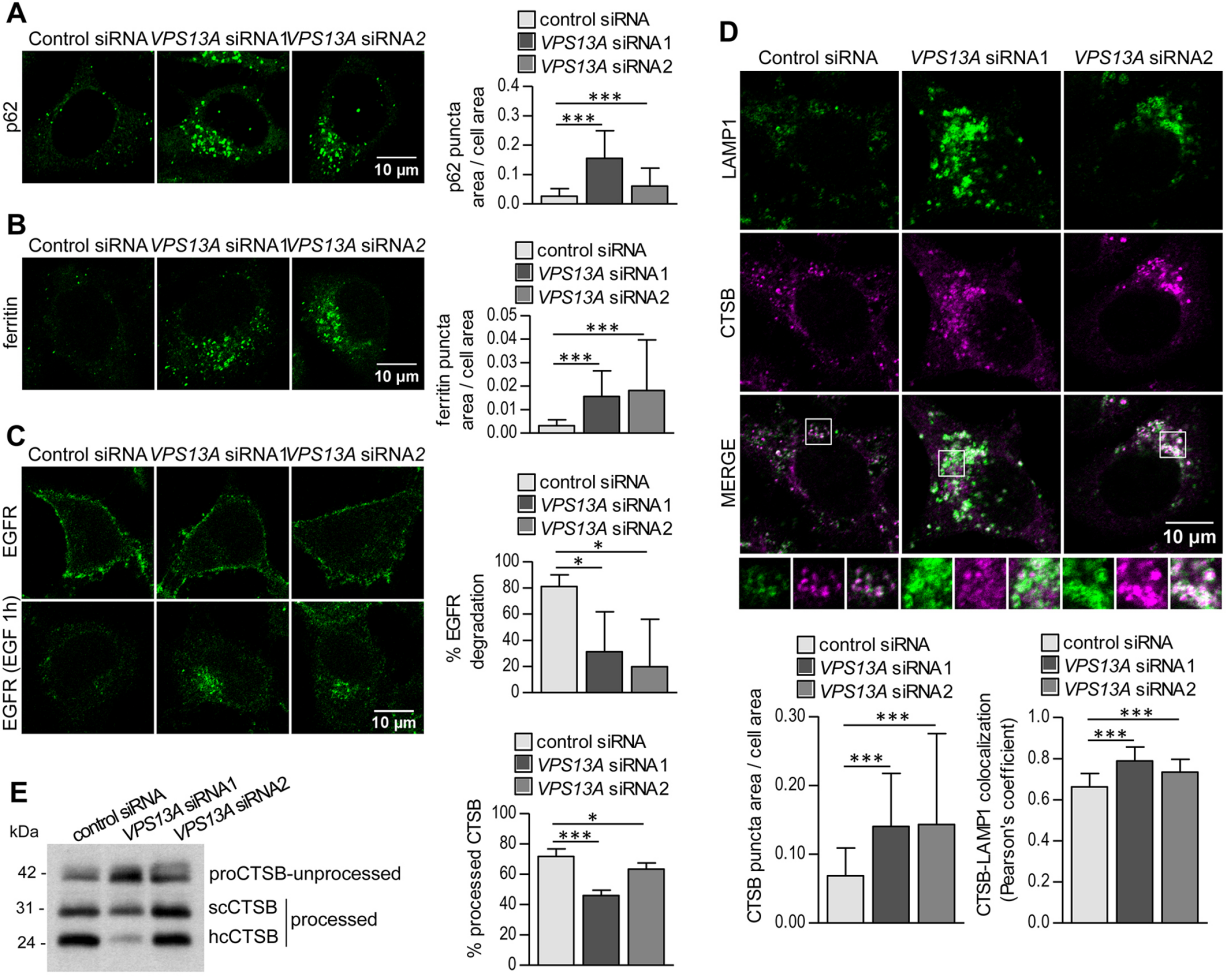
**Lysosomal degradation is reduced in VPS13A-depleted cells.** (A) p62 was observed by immunofluorescence of HeLa cells previously treated with control or *VPS13A* siRNAs in three independent experiments. p62 puncta were accumulated in the absence of VPS13A as quantified in 20 cells per condition and experiment. (B) Immunofluorescence of ferritin was performed in control or *VPS13A* siRNA-treated HeLa cells. In three independent experiments, ferritin-positive puncta accumulated, as observed by confocal microscopy and measured by the area of the puncta per total cell area, at an average of 18 cells per condition and experiment. (C) EGFR was visualized by immunofluorescence of siRNA-treated HeLa cells previously treated with or without EGF, which provokes the internalization and degradation of EGFR in the lysosomes. The remaining signal in an average of 19 cells per condition and experiment in three independent experiments was quantified, and the percentage of degraded EGFR was calculated. (D) CTSB accumulation and colocalization with LAMP1 were observed by immunofluorescence in *VPS13A* siRNA-treated cells and quantified in 14 cells, on average, per condition and experiment in a total of three independent experiments. Enlargements of selected areas are shown. (E) The amount of unprocessed pro-CTSB (proCTSB) and processed single-chain (scCTSB) and heavy-chain (hcCTSB) forms of CTSB in lysates of HeLa cells treated with control or *VPS13A* siRNAs was analyzed by western blotting in three independent experiments, and the percentage of total CTSB that was processed was calculated. Means±s.d. are plotted (*P<0.05, ***P<0.001).

Our results suggest that loss of VPS13A leads to a compromised ability of cells to degrade cargos contained into autophagic and endocytic vesicles. To test this hypothesis, we studied the levels of specific cargos that reach the lysosomes through autophagy or endocytosis. p62 (also known as SQSTM1) binds ubiquitinated cargos and LC3 and allows engulfment of cargo by autophagosomes (Pankiv et al., 2007). We observed that the knockdown of *VPS13A* led to a juxtanuclear accumulation of p62 (Fig. 4A), thus supporting the hypothesis of a deficient degradation. Ferritin is another autophagic cargo (Ott et al., 2016), and we also observed its accumulation in *VPS13A* siRNA-treated cells (Fig. 4B). Epidermal growth factor receptor (EGFR) is internalized upon EGF binding, traffics along the endocytic pathway and is degraded in lysosomes (Futter et al., 1996). We visualized the receptor after EGF stimulation and found that, although most EGFR disappeared 1 h after EGF addition in control siRNA-treated cells, internalized EGFR was still present and accumulated in the juxtanuclear region when *VPS13A* was knocked down (Fig. 4C). Together, these results strongly support our hypothesis that VPS13A is necessary for efficient lysosomal degradation.

Lysosomal degradation is carried out by lysosomal hydrolases, which are synthesized as proenzymes, bound to mannose-6-phosphate receptors, and delivered to the lysosomes. In the acidic environment of lysosomes, proenzymes are released from their receptors and proteolytically processed into active forms (Bonifacino and Rojas, 2006; Ishidoh and Kominami, 2002). Considering the described role of yeast Vps13 in vesicular trafficking (Brickner and Fuller, 1997; De et al., 2017; Redding et al., 1996), it was tempting to speculate that VPS13A was necessary in the transport of proenzymes to the lysosomes. To study this possibility, the localization of the lysosomal cysteine protease cathepsin B (CTSB), an example of lysosomal hydrolase, was analyzed. We found that CTSB accumulated in the juxtanuclear area and colocalized with LAMP1 in VPS13A-depleted cells (Fig. 4D), indicating that there is not a major impairment of CTSB delivery to lysosomes. Lysosomal hydrolases such as CTSB may be properly delivered to lysosomes but may not be fully active, as activation requires the proteolytic processing of the enzyme. In the case of CTSB, the cleavage of its propeptide generates an active single-chain protease and this mature form can be further processed into a double-chain form by an additional cleavage that generates a heavy chain and a light chain (Ishidoh and Kominami, 2002). CTSB processing was analyzed by western blotting and found to be decreased in the absence of VPS13A (Fig. 4E). This further supports the idea that VPS13A is necessary for efficient lysosomal degradation, although further research is needed to determine the mechanism by which loss of VPS13A leads to reduced CTSB processing.

### VPS13A largely colocalizes with mitochondria

**Fig. 5.**
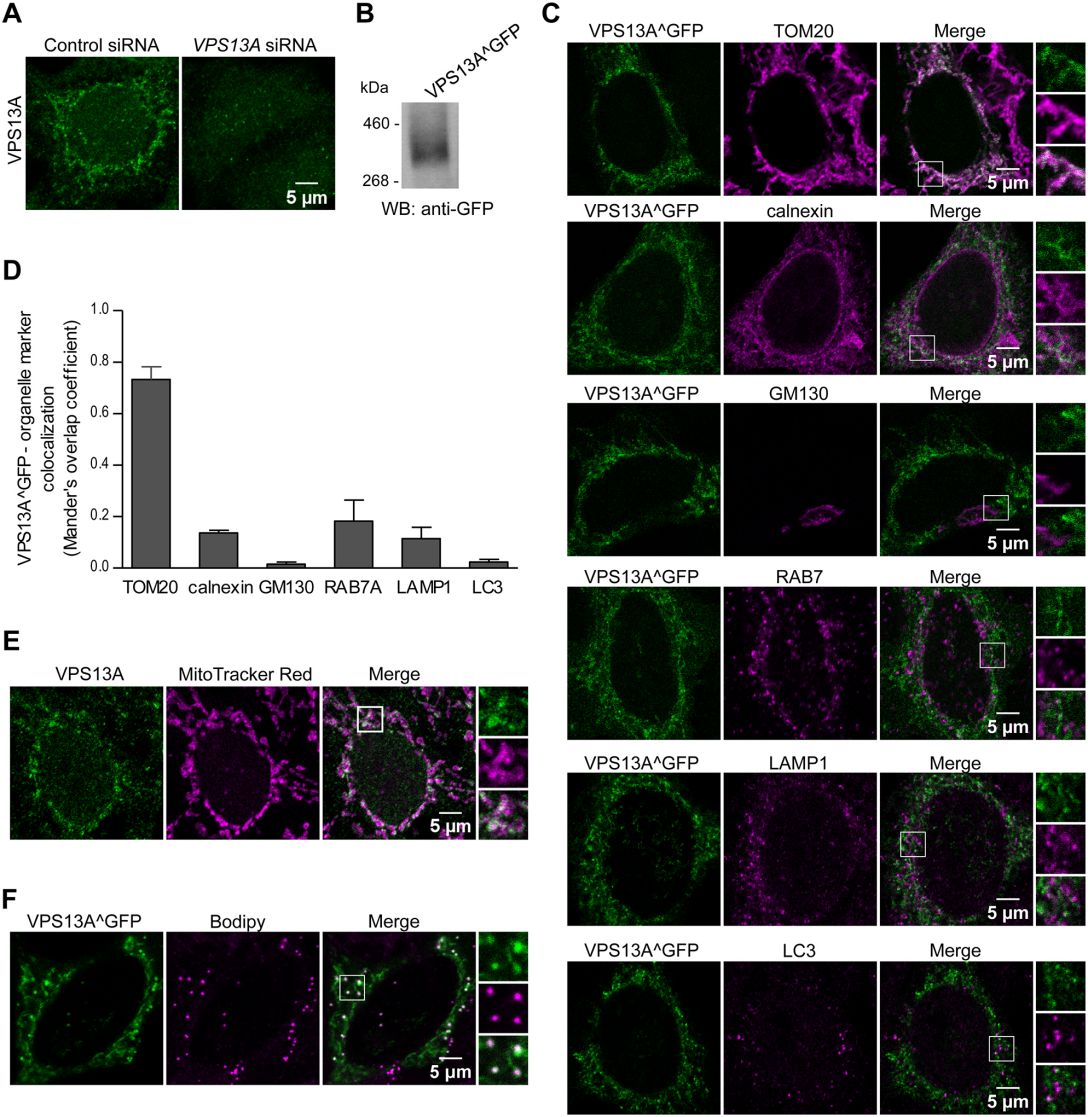
**VPS13A preferentially colocalizes with mitochondria.** (A) Endogenous VPS13A in HeLa cells was labeled by immunofluorescence and visualized by confocal microscopy. VPS13A presents a distinct subcellular network-like pattern that disappears upon treatment with *VPS13A* siRNAs. Images are representative of three independent experiments. (B) VPS13A was tagged with GFP at an internal position and lysates of HeLa cells transiently expressing the resulting VPS13A^GFP protein were analyzed by western blotting using an anti-GFP antibody to verify that the tagged protein had the expected molecular size. (C) Transiently transfected VPS13A^GFP HeLa cells were fixed and labeled with antibodies against TOM20 (also known as TOMM20), calnexin, GM130 (also known as GOLGA2), RAB7A, LAMP1 or LC3B (markers of mitochondria, the ER, *cis*-Golgi, endosomes, lysosomes or autophagosomes, respectively). Examples of VPS13A^GFP puncta colocalizing with those markers are enlarged in the selected areas. Representative images of two independent experiments are shown. (D) Quantification of the colocalization of VPS13A with the above-mentioned organelle markers from an average of 13 cells per marker. The means±s.d. of Mander's overlap coefficient for VPS13A after thresholding are plotted. (E) Endogenous VPS13A was labeled by immunofluorescence and visualized in HeLa cells previously treated with Mitotracker Red CMX ROS to stain mitochondria. (F) Transiently transfected VPS13A^GFP HeLa cells were treated with BODIPY to stain lipid droplets. Enlargements of selected areas are shown to better visualize the colocalization between VPS13A and the organelle markers.

To shed light on how the lack of VPS13A negatively affects cell competence for lysosomal degradation, we investigated the subcellular localization of the protein. Immunofluorescence analysis of endogenous VPS13A revealed a faint signal distributed in a network pattern that disappeared in VPS13A-depleted cells, confirming the specificity of the antibody (Fig. 5A). Because the signal detected by immunofluorescence was low, probably due to the low abundance of the endogenous protein, we set out to overexpress and visualize a GFP-tagged version of VPS13A to continue the analysis of the subcellular localization of the protein. Lang and colleagues had previously reported that yeast Vps13 was not fully functional when tagged at the N or C terminus. However, GFP insertion at a specific internal location did not affect the function of the protein (Lang et al., 2015). The need to place the GFP in an internal position was later confirmed by Park and colleagues, who obtained similar results using an insertion of the GFP at a different position (Park et al., 2016a). We generated, by gap repair, an expression plasmid for VPS13A in which EGFP was inserted in-frame into the human VPS13A sequence after alanine 476 (the equivalent position to that of yeast Vps13 reported by Lang and colleagues). The gap repair strategy to obtain this plasmid is detailed in the Materials and Methods section and depicted in Fig. S2. Overexpressed VPS13A^GFP had the expected molecular weight (Fig. 5B) and presented a network-like pattern similar to that observed for endogenous VPS13A (Fig. 5C). Then, we tested the colocalization of VPS13A^GFP with different organelle markers and found that most VPS13A^GFP clearly decorated the mitochondrial network. We also observed a partial colocalization of VPS13A^GFP with the ER and no colocalization with the Golgi apparatus. To our surprise, we detected only occasional and partial colocalization of VPS13A^GFP with RAB7A and merely sporadic overlap with LC3 and LAMP1 (Fig. 5C,D). The major localization to the mitochondria, partial colocalization with the ER, endosomes and lysosomes, and scarce or no colocalization with the Golgi apparatus were confirmed by detection of endogenous VPS13A by immunofluorescence (Fig. 5E; Fig. S3). Additionally, VPS13A^GFP was observed to colocalize with lipid droplets, according to BODIPY staining (Fig. 5F).

### VPS13A localizes in overlapping regions of the mitochondria and other organelles

**Fig. 6.**
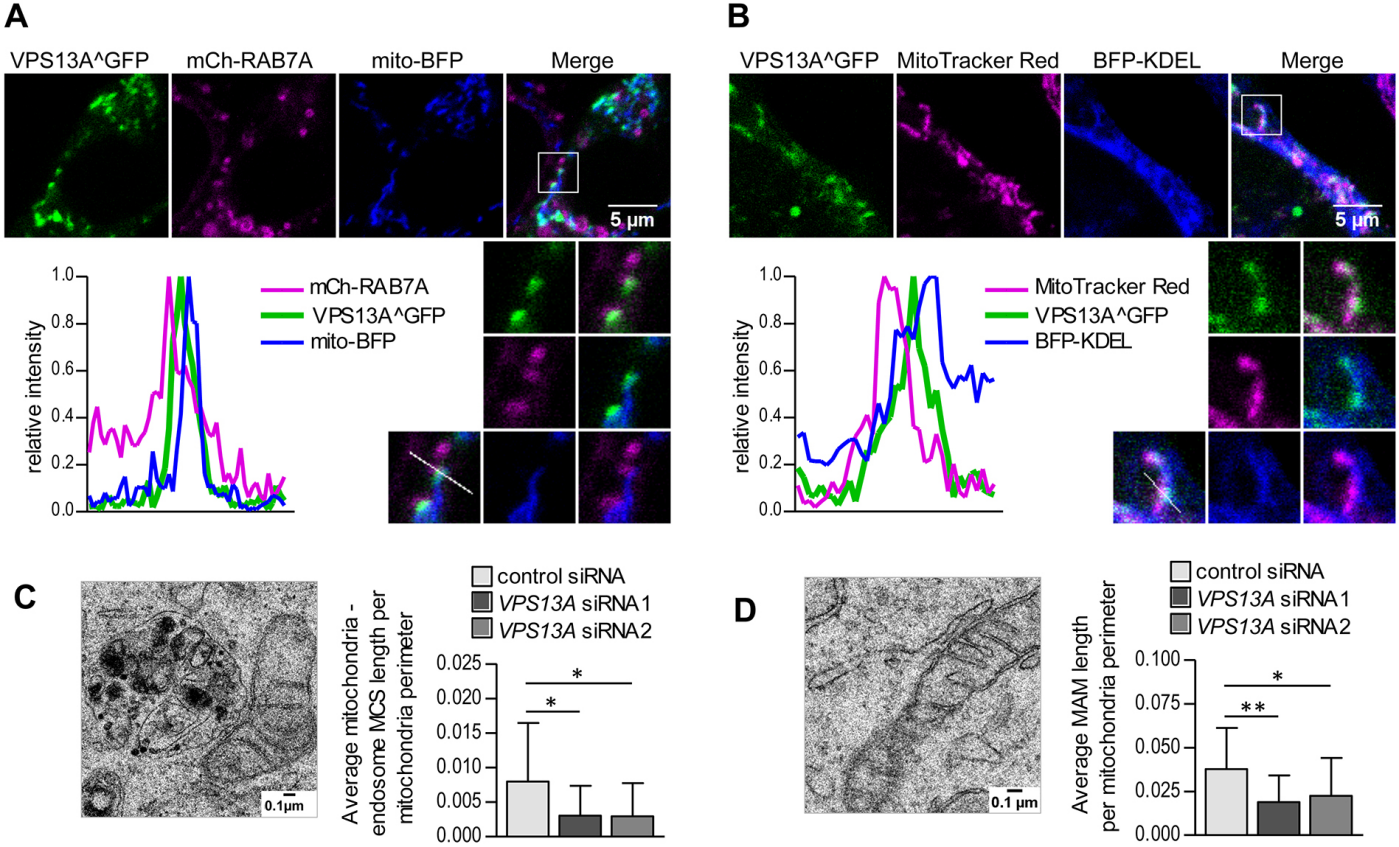
**VPS13A localizes in regions in which mitochondria overlap with other organelles and can influence the length of mitochondria in contact with other organelles.** (A,B) VPS13A^GFP was visualized in cells transiently transfected with mCherry-RAB7A and mito-BFP to label endosomes and mitochondria (A) or cells transiently transfected with BFP-KDEL and stained with Mitotracker Red CMX ROS to label the ER and mitochondria (B). Images are representative of 10 cells. Enlargements of regions in which mitochondria overlap with endosomes (A) or the ER (B) are also shown. For a better visualization of VPS13A localization at the interface between organelles, the plots represent the relative intensity of the fluorescent signal for each marker along the depicted lines in the enlarged regions. (C,D) HeLa cells treated with control or *VPS13A* siRNAs were observed by TEM. MCS between mitochondria and endosomes (C) and between mitochondria and the ER (D) were identified (an example of each type of MCS is shown) and their average length per mitochondrial perimeter was measured in 17 random cell sections per sample. MAM, mitochondria-associated ER membrane. Means±s.d. are plotted (*P<0.05, **P<0.01).

In view of the above results, and based on the studies on yeast Vps13 that demonstrated the localization of the protein at the MCS between the mitochondria and the vacuole or endosomes (Lang et al., 2015; Park et al., 2016a), we wanted to determine whether VPS13A could also be present at the interface between mitochondria and endolysosomes. Triple imaging of VPS13A, mitochondria and RAB7A showed that ∼3% of VPS13A^GFP overlapped simultaneously with mitochondria and RAB7A-positive vesicles, suggesting that the human VPS13A protein might similarly localize at MCS between mitochondria and endolysosomes (Fig. 6A). In addition, we observed that, on average, 12% of VPS13A^GFP overlapped with both the mitochondria and the ER, indicating that VPS13A may also be present in the MCS between the mitochondria and the ER (Fig. 6B). The visualization of VPS13A localization by immunoelectron microscopy would be useful to confirm the association of the protein with mitochondria at contact sites with other organelles. However, to date, the low transfection efficiency of the overexpressed protein and the modest signal obtained with the anti-VPS13A antibody make this task challenging.

Our observations on VPS13A localization at mitochondria-endolysosome and mitochondria-ER MCS (MAM) led us to examine whether the lack of VPS13A had an impact on these MCS. By using TEM, we found endosomal vesicles occasionally adjacent to mitochondria (at a distance of less than 30 nm) in both control and *VPS13A* siRNA-treated cells. The length of those MCS per mitochondria length was reduced upon *VPS13A* knockdown (Fig. 6C); however, the formation of the contacts was not hampered, indicating that the loss of VPS13A does not have a major impact on their formation. With respect to the mitochondria-ER MCS or mitochondria-associated ER membranes, we also found a decrease in the relative length of the contacts (Fig. 6D). The study of the mitochondria-endolysosome MCS suggests that the impaired degradative capacity of lysosomes observed in the absence of VPS13A, a protein with a mitochondrial localization, is not caused by the lack of a physical connection between mitochondria and lysosomes, although the absence of VPS13A may lead to a moderate decrease in the formation or stabilization of these MCS and, possibly, to their functional impairment.

## DISCUSSION

In this study, we have shown that the absence of VPS13A, a condition that leads to ChAc in humans, results in defective lysosomal degradation in HeLa cells. This is probably the cause of the partially impaired autophagy reported in our previous work (Muñoz-Braceras et al., 2015) and the accumulation of non-degraded material found in this study. This defect can potentially lead to the presence of protein aggregates in ChAc, as in many other neurodegenerative diseases. Although no protein aggregates have, as yet, been detected in neuropathologic samples (Walker, 2015b), potential protein aggregates have been visualized at the ultrastructural level in the skeletal muscle of patients with ChAc (Melone et al., 2002). Moreover, p62 is accumulated in ChAc red blood cells, where remnants of vesicles, mitochondria and lysosomes are also indicative of delayed autophagic clearance, at least during erythroid maturation (Lupo et al., 2016). VPS13A is also likely to participate in autophagic clearance in brain neurons, because overexpression of human VPS13A was able to reduce the accumulation of Ref(2)P (the *D. melanogaster* p62 homolog) and ubiquitin-positive aggregates in the central nervous system of a fly mutant for the *VPS13A* homolog (Vonk et al., 2017).

Although this is the first time that an interaction of RAB7A with human VPS13A and with a *D. discoideum* VPS13 protein has been demonstrated, the interaction of other members of the VPS13 family with different RAB GTPases has already been reported. The interaction of VPS13C with diverse RAB GTPases, including RAB7A, has been revealed by different proteomic studies (Behrends et al., 2010; Huttlin et al., 2015; McCray et al., 2010), and VPS13B has been described as an effector of RAB6 (also known as RAB6A) (Seifert et al., 2015). In addition, the three *D. melanogaster* VPS13 proteins were isolated by affinity chromatography using different GTP-RABs (Gillingham et al., 2014). RAB7A is involved in many different stages of endosomal and autophagosomal trafficking, lysosomal fusion and lysosomal maintenance, among other processes (reviewed by Guerra and Bucci, 2016). Defects in any of these processes can lead to defective lysosomal degradation. It is therefore conceivable that VPS13A may play a role in autophagy and in the endolysosomal pathway through its interaction with RAB7A. Alternatively, Lupo and colleagues have reported that VPS13A co-immunoprecipitates with ATG7, which may indicate a more direct role of VPS13A in the autophagic pathway (Lupo et al., 2016). However, we found that the absence of VPS13A leads to the accumulation of vesicles with partially degraded content but not to the accumulation of immature autophagosomes, a phenotype that would be expected for a defect in the early stages of autophagosome formation.

Given the requirement of VPS13A for the degradative function of lysosomes and its interaction with RAB7A, it was surprising to observe that the protein only occasionally colocalizes with endolysosomal compartments. Instead, VPS13A mainly colocalizes with mitochondria. Recently, RAB7A, which has been widely characterized as an endosomal marker, has been shown to be present on other endomembranes, including mitochondria, although at low levels (Jimenez-Orgaz et al., 2018). We have not found an evident colocalization of VPS13A and RAB7A along mitochondrial membranes. However, as it has been reported that the visualization of RAB7A at mitochondria requires a high laser power, we cannot rule out that a small amount of RAB7A, undetectable with our confocal settings, is present at mitochondria together with VPS13A. In contrast, we have clearly observed the occasional colocalization of VPS13A with RAB7A in discrete locations corresponding mainly to the overlap of VPS13A in mitochondria with RAB7A-positive vesicles. This observation raises the possibility that VPS13A interaction with RAB7A takes place locally on mitochondrial membranes in contact with endolysosomal vesicles. The restricted contact between these organelles would explain the low but reproducible proportion of the total VPS13A and RAB7A that colocalize and interact with each other. In line with this, a recent report suggests that RAB7A cycling is regulated specifically at mitochondria-lysosome contacts (Wong et al., 2018); and *S.* *cerevisiae* Vps13 can localize at MCS between the mitochondria and endosomes [known as endosome–mitochondria junctions (EMJs)], and between the mitochondria and the vacuole [known as vacuole-mitochondria patches (vCLAMPs)] (Lang et al., 2015; Park et al., 2016a). In addition to VPS13A colocalization with both mitochondria and RAB7A-positive endolysosomes, we detected VPS13A at mitochondria in close proximity to the ER, suggesting that VPS13A can also be present at multiple MCS in mammalian cells. Our results are in agreement with a recent report, published during the revision of this work, which shows VPS13A localization at the junctions between mitochondria and ER tubules (Kumar et al., 2018). Similarly, VPS13A colocalization with lipid droplets was also reported by Kumar et al., but they did not describe the presence of VPS13A at MCS between mitochondria and endosomes. The percentage of lysosomes in contact with mitochondria at any given time is small and the contacts are very dynamic, as further described recently (Wong et al., 2018). Therefore, it is difficult to visualize the contacts between mitochondria and endolysosomes. This might explain the small percentage of VPS13A that overlaps with both mitochondria and endolysosomes that we have described here and the lack of mention of a similar observation by Kumar et al. in their study (Kumar et al., 2018).

Although our analysis by TEM of MCS between mitochondria and endolysosomes, or between mitochondria and the ER, suggests that VPS13A is not essential for their formation, the observed reduction of the length of mitochondria in contact with endosomes or with the ER in *VPS13A* siRNA-treated cells may indicate that VPS13A is involved in the stabilization or extension of the contacts. Our observation on mitochondria-ER MCS as a consequence of *VPS13A* knockdown complements that of Kumar and colleagues, consisting of an expansion of those contacts upon VPS13A overexpression (Kumar et al., 2018). Regarding the contacts between mitochondria and endolysosomes, we speculate that a role of VPS13A in these MCS would explain how the loss of mitochondria-localized VPS13A can result in the alteration of the endolysosomal pathways. The function of the MCS between mitochondria and endolysosomes is currently unclear. In yeast, their formation can compensate for the absence of ERMES and, interestingly, Vps13 is required for this effect (Lang et al., 2015; Park et al., 2016a). Studies in yeast suggest that the MCS between the mitochondria and endosomes or the vacuole, like those between the mitochondria and the ER, may be involved in the trafficking of lipids, ions or other metabolites to and from the mitochondria (Elbaz-Alon et al., 2014; Hönscher et al., 2014); and, interestingly, the recent report by Kumar and colleagues showed that the N-terminal region of *S.* *cerevisiae* Vps13 can transfer lipids, raising the possibility that VPS13A may be required for lipid trafficking at MCS (Kumar et al., 2018). In mammalian cells, the MCS between mitochondria and endosomes have only recently been described. To date, they have been implicated in iron transfer (Das et al., 2016; Hamdi et al., 2016), mitochondrial fission (Hsu et al., 2018; Wong et al., 2018), and lysosomal dynamics and RAB7A hydrolysis (Wong et al., 2018). Therefore, a hypothetical role of VPS13A in the MCS between mitochondria and endolysosomes may be required for a correct local regulation of RAB7A, leading to the observed changes in RAB7A levels and localization, and could potentially explain the alteration of lysosomal function when VPS13A is absent.

Although loss of VPS13A function at MCS between mitochondria and endolysosomes is the most direct explanation of the defects observed upon depletion of the mitochondria-localized VPS13A protein, we cannot rule out an alteration of a more indirect crosstalk between organelles as the cause of the endolysosomal dysfunction. Many cases have been reported in which the dysregulation of one organelle affects the activity of the other (Raimundo et al., 2016; Soto-Heredero et al., 2017). As a consequence of this functional connection, both mitochondrial and lysosomal homeostasis are often compromised in neurodegenerative disorders, including Parkinson's disease, Alzheimer's disease and lysosomal storage disorders (Audano et al., 2018; Plotegher and Duchen, 2017). Interestingly, defects in the morphology and trafficking of mitochondria and lysosomes have recently been described in medium spiny neurons derived from ChAc patient induced pluripotent stem cells (Glaß et al., 2018). These findings suggest that the mechanism of crosstalk between mitochondria and lysosomes that we describe in HeLa cells may also be relevant in the cells affected in the disease, a possibility that warrants further investigation.

The described endolysosomal dysfunction leading to the accumulation of vesicles with non-degraded material in *VPS13A* knockdown cells may be clinically relevant, as the modulation of lysosomal-related processes (such as autophagy, lysosome biogenesis or lysosomal degradation) is considered a promising potential therapeutic approach for many neurodegenerative diseases with compromised endolysosomal function (Morel et al., 2017; Sardiello, 2016). In conclusion, our findings provide the basis for the study of lysosomes and their communication with mitochondria in the context of ChAc, but further research is necessary to shed more light on how the absence of VPS13A, a protein with a predominant mitochondrial localization, has a negative impact on lysosomal degradation.

## MATERIALS AND METHODS

### Cells, plasmids and antibodies

*Dictyostelium discoideum* original *tipC*^−^ mutant was obtained from Dicty Stock Center, kindly deposited to the repository by William F. Loomis (Stege et al., 1999). The identity of the strain was confirmed by PCR using diagnostic amplifications of the *tipC* disrupted gene. The *tipC*^−^ strains overexpressing GFP alone or the C-terminal region of TipC fused to GFP were generated by electroporation of the pDV-CGFP-CTAP vector itself (GenBank Accession Number EF028672) or a construct containing the DNA genomic region encoding amino acids 2725-3848 of TipC cloned into the pDV-CGFP-CTAP vector, and subsequent selection of the transformed cells as previously described (Muñoz-Braceras et al., 2015). HeLa cells were a gift from Alberto Muñoz from the Biomedical Research Institute, Madrid, Spain, and they were authenticated and tested routinely for contamination.

HA-Rab7A plasmid was generated by PCR amplification of *D. discoideum* complementary DNA (cDNA), using the oligonucleotides ddRAB7-Fw and ddRAB7-Rv (Table S2), and cloned into the pDM358 extrachromosomal vector (GenBank Accession Number EU912542) (Veltman et al., 2009). The VPS13A^EGFP plasmid was generated by a complex strategy including conventional cloning and a gap repair approach and hence it is described in detail later in the Materials and Methods section. GFP-RAB7A plasmids were a gift from Patricia Boya from the Biological Research Center of Madrid. The rest of the plasmids were purchased from Addgene: mCherry-RAB7A (plasmid 61804), mito-BFP (plasmid 49151) and BFP-KDEL (plasmid 49150). The sequences of all plasmids were confirmed by Sanger sequencing.

Primary antibodies used in this study were as follows: anti-calnexin [BD Transduction Laboratories, 610523; used at 1:100 for immunofluorescence (IF)], anti-CTSB [RyD Systems, AF953; 1:1000 for western blotting (WB) and 1:150 for IF], anti-EGFR (Cell Signaling Technology, 4267; 1:100 for IF), anti-GAPDH (Enzo LifeSciences, ADI-CSA-335; 1:2000 for WB), anti-ferritin (RyD Systems, MAB93541; 1:100 for IF), anti-GFP (Sigma-Aldrich, G1544; 1:4000 for WB), anti-GM130 (BD Transduction Laboratories, 610823; 1:100 for IF), anti-HA (Sigma-Aldrich, 12CA5; 1:1000 for WB), anti-LAMP1 (Cell Signaling Technology, 15665; 1:100 for IF), anti-LC3B (Cell Signaling Technology, 2775; 1:200 for IF; Nanotools, 0260-100/LC3-2G6; 1:100 for IF;), anti-RAB7A (Cell Signaling Technology, 9367; 1:100 for IF and 1:1000 for WB; Abcam, ab137029; 1:100 for IF), anti-RILP (Novus, NBP1-98278; 1:600 for WB), anti-p62 (Epitomics, 3340-1; 1:150 for IF), anti-TOM20 (Santa Cruz Biotechnology, sc-11415; 1:100 for IF) and anti-VPS13A (Sigma-Aldrich, HPA021662; 1:75 for IF and 1:500 for WB). Alexa-Fluor-dye-conjugated secondary antibodies were purchased from Invitrogen and used at 1:500 for IF and horseradish-peroxidase-conjugated secondary antibodies were purchased from Santa Cruz Biotechnology and used at 1:5000 for WB.

### Construction of the VPS13A^EGFP expression plasmid

The plasmid for the expression of VPS13A with EGFP inserted in-frame after alanine 476 was constructed by gap repair, which takes advantage of the efficient homologous recombination in yeast. A scheme of the strategy to generate the plasmid is shown in Fig. S2 and described in detail below. Overlapping fragments of VPS13A transcript variant A (NM_033305) were obtained by PCR amplification of cDNA from HeLa cells, EGFP was amplified from the pEGFP-C3 plasmid, and the ori (2 μm) and URA3 elements, necessary for plasmid selection and amplification in yeast, were amplified from the pRS426 plasmid. The corresponding oligonucleotide sequences are shown in Table S3. A pEGFP-C3 plasmid without the EGFP sequence (between the *Age*I and *Sca*I restriction sites) was generated and used as a backbone for gap repair after digestion with the *Ase*I and *Xho*I restriction enzymes. The PCR products and the vector fragments were transformed in the *S. cerevisiae* strain FY250 for their assembly by gap repair as previously described (Joska et al., 2014). A plasmid of 17 kb was generated, isolated and transformed in DH5α *Escherichia coli* by a conventional heat shock procedure to be amplified. To reduce the size of the plasmid, we cloned the 11 kb fragment between the *Acl*I and *Mlu*I restriction sites into the *Cla*I and *Btg*I sites of the pBluescript SK vector and a final VPS13A^EGFP expression plasmid of 14 kb was obtained. The whole VPS13A^EGFP sequence was verified by Sanger sequencing.

### Cell culture and treatments

*Dictyostelium discoideum* cells were grown axenically in liquid non-defined HL-5 complete medium (Formedium, HLB0102) supplemented with 0.014 g/ml glucose (Pronadisa, 2150) and 25 U/ml penicillin and 0.025 mg/ml streptomycin (Sigma-Aldrich, P4458) in an incubator at 22°C. HeLa cells were grown in complete Dulbecco's modified Eagle medium (DMEM; Sigma-Aldrich, D5648), supplemented with 10% fetal bovine serum (FBS; Gibco, 10500064) and 50 U/ml penicillin and 0.05 mg/ml streptomycin, in standard culture conditions (37°C, 5% CO_2_).

For EGFR degradation assay, cells were incubated overnight in serum-free DMEM and then treated with 100 ng/ml EGF (Sigma-Aldrich, E9644) for the indicated times. Staining of mitochondria was performed by incubating the cells with 100 nM MitoTracker Red CMXRos (Molecular Probes, M7512) for 15 min and lipid droplets were stained using 1 μg/ml BODIPY 558/568-C_12_ (Molecular Probes, D3835) for 30 min. In all cases, cells were incubated in standard culture conditions during treatment with these compounds.

### DNA and siRNA transfection

DNA transfection was performed using Lipofectamine 2000 (Invitrogen, 11668019), according to the manufacturer's protocol. Briefly, Lipofectamine 2000 and DNA were mixed in Opti-MEM reduced serum medium (Life Technologies, 31985062) and DNA-Lipofectamine 2000 complexes were added to the cells. Cells were visualized 24 h after the DNA transfection. *VPS13A* expression was inhibited by reverse transfection of validated siRNAs (Ambion, siRNA ID s23340, named as *VPS13A* siRNA1; or siRNA ID s23342, named as *VPS13A* siRNA2). Silencer select negative control siRNA (Ambion, ID No. 2 4390846) was used to transfect cells as a control of the knockdown experiments. An additional control siRNA (Ambion, ID No. 1 4390843) was used to confirm that the phenotypes observed with several markers (p62, LAMP1, RAB7A and LC3) were not affected by the used siRNA control (data not shown). Transfection of siRNA was performed using Lipofectamine RNAiMAX (Invitrogen, 13778030) according to the manufacturer's instructions. Briefly, siRNA and Lipofectamine RNAiMAX were mixed in Opti-MEM and cells were added to the mixture. Knockdown cells were transfected again 48 h after the first transfection with the same siRNAs and the experiments were carried out 72 h after the second transfection. The level of *VPS13A* mRNA was measured by TaqMan PCR (Applied Biosystems; VPS13A, Hs00362891_m1; endogenous control gene used for normalization, Hs00183533_m1). Both *VPS13A* siRNAs significantly decreased *VPS13A* mRNA levels, although the inhibition of the expression was stronger using the *VPS13A* siRNA1 (80.0±16.5% for siRNA1 and 53.0±8.0% for siRNA2). The levels of *VPS13C* mRNA were not altered by any of the *VPS13A* siRNAs as determined by TaqMan PCR (Applied Biosystems; VPS13C, Hs00419559_m1).

### Immunoprecipitation assays

GFP-tagged proteins were immunoprecipitated with GFP-Trap A beads (Chromoteck, gtak-20) according to the manufacturer's protocol. Briefly, *D. discoideum* or HeLa cells expressing the GFP-tagged protein were washed with PDF buffer (20 mM KCl, 9 mM K_2_HPO_4_, 13 mM KH_2_PO_4_, 1 mM CaCl_2_, 1 mM MgSO_4_, pH 6.4) or phosphate-buffered saline (PBS) 1× (133 mM NaCl, 8 mM Na_2_HPO_4_, 2 mM KH_2_PO_4_, pH 7.4), respectively, harvested and lysed using protein lysis buffer (10 mM Tris-HCl, pH 7.5, 150 mM NaCl, 0.5% NP-40), supplemented with phosphatase inhibitors (2.5 mM NaF, 0.2 mM Na_3_VO_4_) and 1:100 protease inhibitor cocktail (Sigma-Aldrich, P8340). Cell lysates were centrifuged (12,000 ***g*** for 15 min at 4°C) and the supernatant was transferred to a microcentrifuge tube. Pre-washed GFP-Trap A beads were added, and the mixture was diluted in washing buffer (10 mM Tris-HCl pH 7.5, 150 mM NaCl, 2.5 mM NaF, 0.2 mM Na_3_VO_4_ and protease inhibitors) and incubated for 4 h at 4°C with continuous rotation. Then, the mixture was centrifuged (2000 ***g*** for 2 min at 4°C) and the beads were washed five times with washing buffer and prepared for mass spectrometry or western blot analyses.

### Sample preparation and protein identification by nano LC–MS/MS QTOF (Triple TOF) analysis

The protein extract samples were resuspended in a volume up to 100 μl of Laemmli sample buffer and applied onto a conventional 4% stacking and 12% resolving SDS-PAGE gel. Electrophoresis was stopped as soon as the front entered 1 cm into the resolving gel in order to concentrate the whole proteome in the stacking/resolving gel interface. The unresolved protein bands were visualized by Coomassie Blue staining and each gel lane was excised, cut into cubes (1 mm^2^), deposited into 96-well plates and automatically processed in a Proteineer DP digestor (Bruker Daltonics). Digestion was performed as previously described (Shevchenko et al., 1996), with the following minor modifications: gel plugs were washed first with 50 mM NH_4_HCO_3_ and then with acetonitrile (ACN) prior to reduction with 10 mM dithiothreitol in 25 mM NH_4_HCO_3_ solution, and alkylation was carried out with 55 mM iodoacetamide in 50 mM NH_4_HCO_3_ solution. Gel pieces were rinsed with 50 mM NH_4_HCO_3_, then with ACN, and dried under a stream of nitrogen. Proteomics Grade Trypsin (Sigma-Aldrich, T6567) at a final concentration of 16 ng/μl in 25% ACN/50 mM NH_4_HCO_3_ solution was added and digestion took place at 37°C for 4 h. The reaction was stopped by adding 50% ACN/0.5% trifluoroacetic acid (TFA) for peptide extraction and the tryptic eluted peptides were dried by speed-vacuum centrifugation. The peptide samples were analyzed on a nano LC system (Eksigent Technologies nanoLC Ultra 1D plus, AB SCIEX) coupled to a 5600 Triple TOF mass spectrometer (AB SCIEX) with a nanoelectrospray ion source. Samples were injected on a C18 PepMap trap column (5 µm, 100 µm ID ×2 cm, Thermo Scientific) at 2 µl/min, in 0.1% formic acid in water, and the trap column was switched on-line to a C18 nanoAcquity BEH analytical column (1.7 µm, 100 Å, 75 µm ID ×15 cm, Waters). Equilibration was performed in mobile phase A (0.1% formic acid in water) and peptide elution was achieved in a 60 min gradient from 5% to 40% B (0.1% formic acid in acetonitrile) at 250 nl/min. The mass spectrometer operated in data-dependent acquisition mode. For TOF scans, the accumulation time was set to 250 ms and, per cycle, up to 15 precursor ions were monitored. MS and MS/MS data obtained for each sample were processed using Analyst TF 1.5.1 Software (AB SCIEX, Foster City, CA, USA). Raw data were translated to mascot general file (mgf) format and searched against a composite target/decoy database built from the 8418 sequences in the *D. discoideum* reference proteome at Uniprot Knowledgebase (as of July 2015), using an in-house Mascot Server v. 2.4 (Matrix Science, London, UK). Search parameters were set as follows: carbamidomethylcysteine as fixed modification and methionine oxidation as variable one. Peptide mass tolerance was set to 30 ppm and 0.05 Da, in MS and MS/MS mode, respectively, and two missed cleavage sites were allowed. False discovery rates (FDR) for peptide identification were calculated and only the peptides passing a cutoff of FDR<1% were taken into account. In addition, absence of identification in the control GFP pulldown sample and a coverage above 10% of the protein were set as the parameters to consider the identified proteins as strong candidates for the interaction. However, 60S and 40S ribosomal proteins were ignored, even if meeting the cited criteria.

### Western blot experiments

For protein extraction, cells were washed with PBS, harvested and lysed with lysis buffer (50 mM Tris-HCl, pH 8, 150 mM NaCl, 1% Triton X-100, supplemented with phosphatase and protease inhibitors) unless otherwise indicated. Cell lysates were centrifuged (12,000 ***g*** for 15 min at 4°C) and the protein concentration of the supernatant was determined using the Pierce BCA Protein Assay Kit (Thermo Fisher Scientific, 23225). Equal amounts of protein samples were prepared in 1× SDS-PAGE sample loading buffer, heated, loaded and run for separation in conventional handcast SDS-polyacrilamide gels. For VPS13A detection, precast NuPage Tris-acetate 3-8% polyacrylamide gradient gels (Invitrogen, EA0378) were used. Electrotransference on Immobilon-P polyvinylidene difluoride (PVDF) membranes (Millipore, IPVH00010) was performed by wet transfer. For immunoblot analysis, membranes were blocked in 5% skimmed milk in TBS-T buffer for at least 1 h at room temperature (RT), incubated with primary antibodies in blocking solution overnight at 4°C, washed with TBS-T, incubated with the appropriate horseradish-peroxidase-conjugated secondary antibodies for 1 h at RT, and washed before incubation with Amersham ECL Western Blotting Detection Reagents (GE Healthcare Life Sciences, RPN2134) or Clarity Western ECL Substrate (Bio-Rad, 1705060) for antibody detection. The chemiluminescent signal was captured using CURIX RP2 Plus films (Agfa), which were introduced in an X-ray film-processing machine and scanned. Intensity of bands was performed by densitometry using ImageJ software (NIH).

### Immunofluorescence staining and confocal microscopy

Cells were grown on glass sterilized coverslips in 24-well plates (Corning, 353047) or in μ-Slide 8-well chambered coverslips (Ibidi, 80826). For immunofluorescence, fixation was performed with 3% paraformaldehyde in PBS, pH 7.4, for 15 min at RT and then washed with PBS and incubated with 100 mM glycine in PBS for 30 min at RT. Permeabilization with cold methanol was performed at −20°C for 10 min. Coverslips were washed with PBS, incubated with blocking solution (3% bovine serum albumin, 0.2% Triton X-100 in PBS) for 1 h at RT, incubated with primary antibodies in blocking solution for 3 h at RT, washed and incubated with the appropriate Alexa-Fluor-dye-conjugated secondary antibodies 1 h at RT. Coverslips were then washed and mounted on slides using Prolong Diamond Antifade Mountant (Molecular Probes, P36970). Images were acquired using an inverted laser confocal microscope (Zeiss, LSM710) with a 63×/1.40 Plan-Apochromatic oil immersion objective and analyzed using ImageJ software.

### TEM

Fixation of cells was performed with 4% paraformaldehyde and 2% glutaraldehyde in 0.1 M phosphate buffer (PB, pH 7.4) for 2 h at RT, followed by a post-fixation with 1% OsO_4_ and 1.5% K_3_Fe(CN)_6_ in water for 1 h at 4°C and dehydration with acetone. *In situ* flat embedding of samples was performed in Epoxy, TAAB 812 Resin (TAAB Laboratories) according to standard protocols. After polymerization, resin sheets were detached from the substrate and mounted onto resin blocks. Ultrathin sections (80 nm) were obtained, deposited onto slot grids and stained with saturated uranyl acetate/lead citrate. Visualization was performed at 80 kV using an electron microscope (Jeol, JEM-1010) and images were recorded with a digital camera (TVIPS, TemCam-F416).

### Image and data analysis

Analysis of images was performed using ImageJ, either manually using tools to select, quantify and measure elements from micrographs or confocal images, or semi-automatically using available plugins such as JACoP for colocalization analyses. Mander's overlap coefficients were calculated using thresholds to define labeled structures. To quantify the simultaneous colocalization of VPS13A with two different organelle markers, a mask with the pixels that were above the threshold in the images of VPS13A and the two organelle markers was created and used to calculate the percentage of VPS13A overlapping with the two organelles simultaneously. Confocal microscopy images were false colored to make them as colorblind friendly as possible.

Data from independent experiments were analyzed using GraphPad Prism (GraphPad Software). Means±s.d. were calculated and represented in plots with error bars. Statistical differences between groups, determined by two-tailed Student's *t-*test, are indicated with asterisks (significant at *P<0.05, **P<0.01, ***P<0.001).

## Supplementary Material

Supplementary information
