# Supplementary material for: VPS13A is closely associated with mitochondria and is required for efficient lysosomal degradation
Source: Dis Model Mech. 2019 Feb 22;12(2):dmm036681. doi: 10.1242/dmm.036681 (PMC6398486; doi:10.1242/dmm.036681)
Supplement: Supplementary information [file dmm-12-036681-s1.pdf]

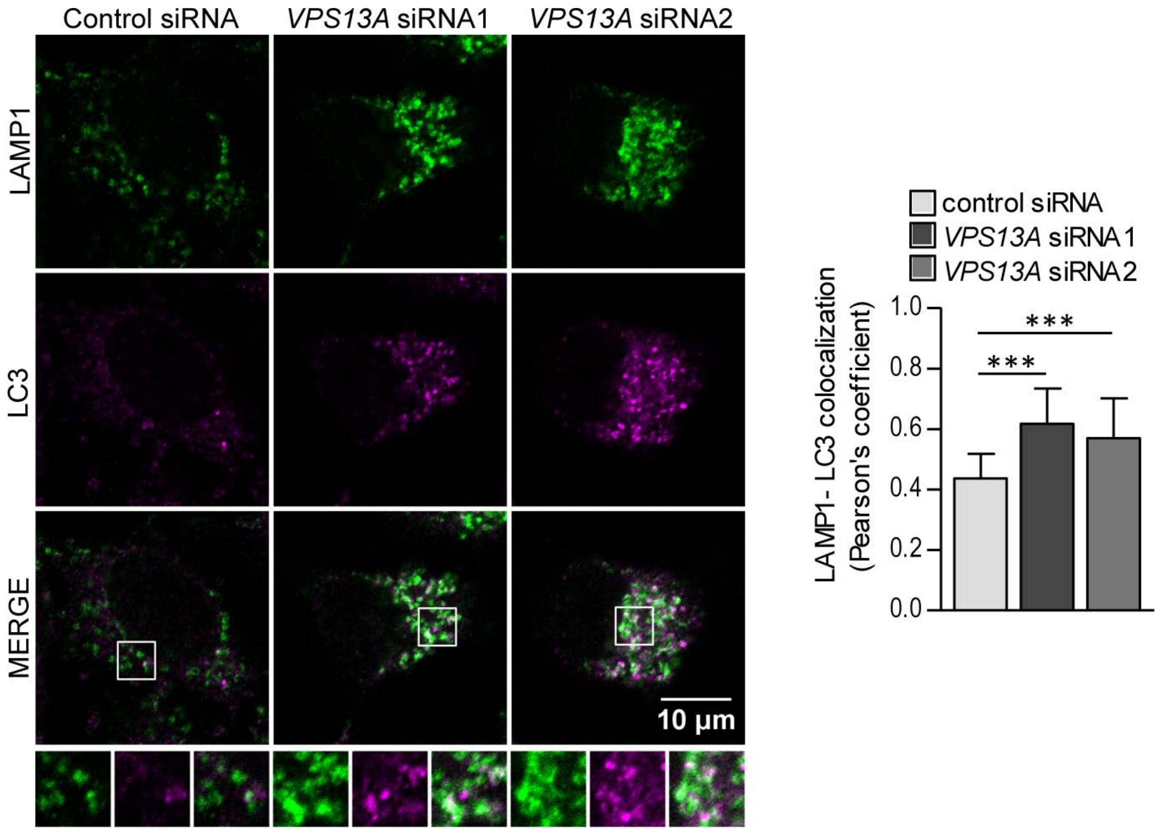

Fig. S1. LAMP1 and LC3 accumulate and colocalize in VPS13A depleted cells. HeLa cells were treated with control or *VPS13A* siRNAs and LAMP1 and LC3 were visualized by immunofluorescence. Representative images, enlargements of selected areas, and the quantification of the LAMP1-LC3 colocalization in 20 cells per condition and experiment are shown. Means ± s.d. of three independent experiments are represented.

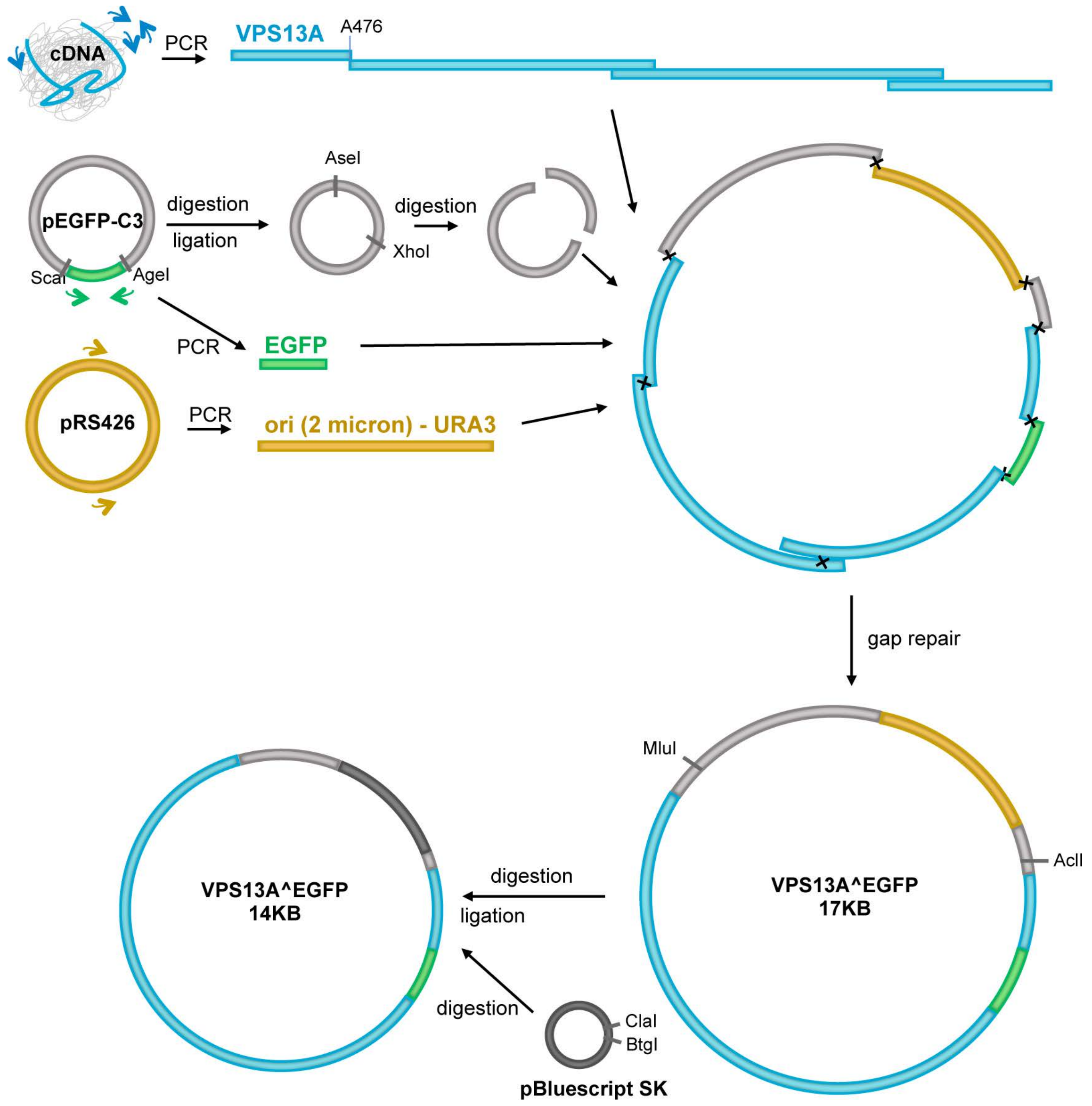

Fig. S2. Scheme of the strategy to construct the VPS13A^EGFP expression plasmid. PCR fragments corresponding to VPS13A cDNA, EGFP, all the elements necessary for plasmid selection and amplification in yeast cells, and two fragments of a digested mammalian expression vector were introduced in *S. cerevisiae* cells, where they were all assembled by homologous recombination. The plasmid generated by this gap repair approach was isolated from yeast cells and amplified in *E. coli* cells transformed with the plasmid. To reduce the size of the construction, a second expression VPS13A^EGFP plasmid was obtained by conventional cloning.

Disease Models & Mechanisms • Supplementary information

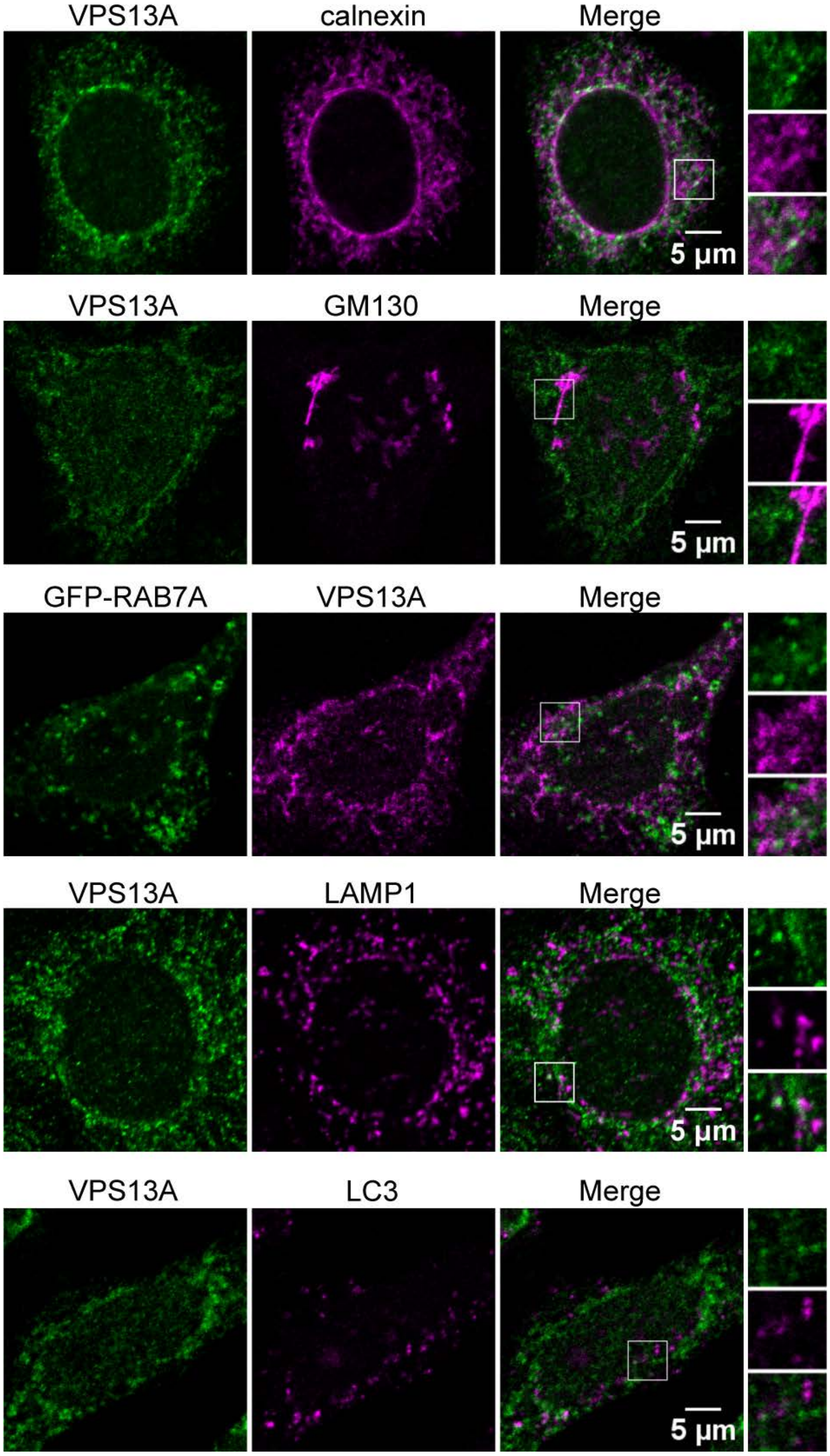

Fig. S3. Endogenous VPS13A colocalization with various organelle markers. Endogenous VPS13A was labeled by immunofluorescence and visualized in HeLa cells labeled with antibodies against calnexin, GM130, LAMP1 or LC3 (markers of the ER, *cis*-Golgi, lysosomes, or autophagosomes, respectively) or transiently transfected with mCherry-RAB7A to visualize endosomes. Enlargements of selected areas are shown to better visualize the colocalization between VPS13A and the organelle markers.

Disease Models & Mechanisms • Supplementary information

Table S1. Proteins identified as strong candidates for the interaction with *D. discoideum* TipC$^{2725\text{-}3848}$-GFP.

UniProt ID	Protein name	Unique peptides	Coverage
Q54GX7	Actin-10	12	46.0
P07828	Actin-18	1	15.0
P0CT31	Elongation factor 1-alpha	10	28.0
P15064	Ras-like protein rasG	4	30.2
P32252	Ras-like protein rasB	2	11.2
P34139	Ras-related protein Rab-1A	4	34.7
Q54NU2	Ras-related protein Rab-1D	2	22.5
P36411	Ras-related protein Rab-7A	3	15.3
P34144	Rho-related protein rac1A	4	21.1
Q54J48	Probable pyridoxal 5'-phosphate synthase subunit pdx2	3	21.8
Q8MPA5	Small heat shock protein hspG7	1	19.8
P14794	Ubiquitin-60S ribosomal protein L40	4	41.4

Table S2. Oligonucleotide primers used during the construction of the HA-Rab7A expression plasmid for *D. discoideum.*

ddRAB7-Fw	5'-GCAAGATCTAAAATGTATCCATATGATGTTCCAGATTATGCAGCCACAAAGAAAAAGGTT-3'
ddRAB7-Rv	5'-CGTACTAGTTTAACAACAACCTGATTTAGC-3'

Table S3. Oligonucleotide primers used during the construction of the VPS13A^EGFP expression plasmid for mammalian cells.

Y-Fw	5'-TTCTGTGGATAACCGTATTACCGCCATGCATTAGTTATGGTTCCGCGCACATTTCCCCG-3'
Y-Rv	5'-TATGAACTAATGACCCCGTAATTGATTACTATTAGTGAGTTTAGTATACATGCATTTAC-3'
EGFP-Fw	5'-GCAGGTGGTGGCGGTTCAGGCGGAGGTGGCTCTGTGAGCAAGGGCGAGGAGCTGTTCACC-3'
EGFP-Rv	5'-GGATCAACCGATCCGCCACCGCCCGAGCCACCGCCACCCTTGTACAGCTCGTCCATGCCG-3'
VPS 13A-1-Fw	5'-TCAGATCCGCTAGCGCTACCGGACTCAGATCGCCACCATGGTTTTCGAGTCGGTGGTCG-3'
VPS13A-1-Rv	5'-AGAGCCACCTCCGCCTGAACCGCCACCACCTGCTGTTTCACTATAGCCAATTGC-3'
VPS13A-2-Fw	5'-GGTGGCGGTGGCTCGGGCGGTGGCGGATCGGTTGATCCAACTTTACTAAAAACATTTG-3'

VPS13A-2-Rv	5'-CCACAACCTCAAGATTTAATGGCAGG-3'
VPS13A-3-Fw	5'-GGATCTACTCCTGCCATTAAATCTTGAGG-3'
VPS13A-3-Rv	5'-CAGACTGTAGATCGGATGTTGTATGG-3'
VPS13A-4-Fw	5'-GTGAAATAGAAGATTCCCTCCCTCC-3'
VPS13A-4-Rv	5'-CGTCGACTGCAGAATTCGAAGCTTGAGCTCGATCAGAGGCTCGGAGAAGGTTCTCTTGC-3'
